# Antibody Drug Conjugates in Glioblastoma – Is There a Future for Them?

**DOI:** 10.3389/fonc.2021.718590

**Published:** 2021-12-03

**Authors:** Sagun Parakh, Joseph Nicolazzo, Andrew M Scott, Hui Kong Gan

**Affiliations:** ^1^Department of Medical Oncology, Austin Hospital, Heidelberg, VIC, Australia; ^2^Tumour Targeting Laboratory, Olivia Newton-John Cancer Research Institute, Heidelberg, VIC, Australia; ^3^School of Cancer Medicine, La Trobe University, Heidelberg, VIC, Australia; ^4^Drug Delivery, Disposition and Dynamics, Monash Institute of Pharmaceutical Sciences, Monash University, Parkville, VIC, Australia; ^5^Department of Medicine, University of Melbourne, Heidelberg, VIC, Australia; ^6^Department of Molecular Imaging and Therapy, Austin Health, Heidelberg, VIC, Australia

**Keywords:** antibody drug conjugates (ADC), glioma, glioblastoma, blood brain barrier, tumour microenvironment, biomarkers, molecular imaging

## Abstract

Glioblastoma (GBM) is an aggressive and fatal malignancy that despite decades of trials has limited therapeutic options. Antibody drug conjugates (ADCs) are composed of a monoclonal antibody which specifically recognizes a cellular surface antigen linked to a cytotoxic payload. ADCs have demonstrated superior efficacy and/or reduced toxicity in a range of haematological and solid tumors resulting in nine ADCs receiving regulatory approval. ADCs have also been explored in patients with brain tumours but with limited success to date. While earlier generations ADCs in glioma patients have had limited success and high toxicity, newer and improved ADCs characterised by low immunogenicity and more effective payloads have shown promise in a range of tumour types. These newer ADCs have also been tested in glioma patients, however, with mixed results. Factors affecting the effectiveness of ADCs to target the CNS include the blood brain barrier which acts as a physical and biochemical barrier, the pro-cancerogenic and immunosuppressive tumor microenvironment and tumour characteristics like tumour volume and antigen expression. In this paper we review the data regarding the ongoing the development of ADCs in glioma patients as well as potential strategies to overcome these barriers to maximise their therapeutic potential.

## Introduction

Glioblastoma (GBM) is an aggressive fatal disease characterised by complex molecular heterogeneity and aggressive infiltrative growth. Despite s decades of trials testing novel agents, the median survival remains unchanged at 14 - 17 months only (1–4). Multiple strategies have been explored with limited success to improve the efficacy of chemotherapy in GBM, including novel formulations, direct administration into the central nervous system (CNS) and targeted vascular disruption; unfortunately, these have often resulted in higher toxicity rates without significantly improving patient outcomes (5–7).

Antibody drug conjugates (ADCs) are a new but proven class of highly potent therapeutics, composed of a monoclonal antibody which specifically recognizes a cellular surface antigen linked to a cytotoxic payload (8). This results in a number of advantages: reduced toxicity due to more targeted delivery of cytotoxic therapy directly into the tumours; enhanced cell kill from the ability of use more toxic drugs that cannot be safely administered systemically; and the additive/synergistic benefit of combined tumour kill from the antibody and the payload respectively (9, 10). The ultimate efficacy of ADCs though relies on the complex interplay between three vital components: antibody, linker and payload. Early failures in the development of ADCs were due in part to challenges associated with these components, however recent advances have resulted in notable successes, resulting in nine ADCs receiving regulatory approval by the Food and Drug Administration in the USA and four ADCs by the European Medicines Agency (8, 11).

ADCs have also been explored for patients with brain tumours but with limited success to date. In particular, the apparent failure of two recent high-profile ADCs has resulted in a lessening of interest to this approach in glioma patients currently (12, 13). In this article, we will review the development of ADCs in glioma patients and summarise the data supporting their on-going development. We will discuss potential strategies to maximise their therapeutic potential by increasing their penetration through the blood-brain barrier (BBB), selection of more biologically relevant targets in the brain and its microenvironment, novel methods of drug targeting, newer payloads and better patient selection.

## Early ADCs in Glioma Therapy

The first generation of ADCs tested in glioma patients comprised mainly immunotoxins and radioimmunotherapy (**Table 1**). Immunotoxins are antibodies conjugated to naturally occurring bacterial toxins, such as *Pseudomonas aeruginosa* exotoxin A and diphtheria toxin. Radioimmunoconjugates utilise isotopes such as iodine-125 or iodine-131 as payloads. These commonly targeted the EGFR axis (either the receptor itself or its mutants and ligands) due the relatively high prevalence of these targets in gliomas and their likely role as an oncogenic pathway in glioma. Targeting the EGFRvIII mutation was particularly attractive. This is comprised of an in-frame deletion of exons 2-7 that results in a truncated by constitutively active receptor (24). Furthermore, the EGFRvIII mutation is relatively frequent (in 20-40% of GBM tumours) but shows a tumour restricted expression pattern compared to wildtype EGFR (24). However, other targets of these early ADCs included IL-13Rα2 receptor, IL4 and transferrin. Unfortunately, these early ADCs were found to be ineffective due to a number of problems including high immunogenicity, unstable linkers, inefficient deliver due *via* early convection delivery systems, biomarker limitations to address tumour heterogeneity and toxicity (25–27).

**Table 1:** Selected ADCs, immunotoxins and radioimmunoconjugates in high grade gliomas.

Drug	Class	Phase	Date	Toxicities	Efficacy	Comments
ADCs i						
ABT-414 (14)	Anti-EGFR ADC with MMAF	I	2015	Lymphopenia, ocular toxicity, brain oedema, increased transaminases	Monotherapy: RR 8%, mOS N/A, PFS-6 24%	Data in EGFR amplified. Phase 2 and 3 studies in progress
					With TMZ: RR 17%, mOS N/A, PFS-6 25%	
AMG-595 (15)	Anti-EGFR ADC with DM1	I	2014	Thrombocytopenia, LFT abnormalities	RR 8%; mOS N/A; PFS-6 N/A	
Immunotoxins						
Cintredexin Besudotoxin (16)	IL13–PE38QQR	III	2010	Pulmonary embolism (8% including one fatal)	RR N/A; mOS 11.3 months; PFS-6 N/A	
NBI-3001 (17)	Circularized IL4–PE38KDEL	I/II	2003	Neurological deficits (Weakness, aphasia, confusion, coma), seizures, headaches, cerebral oedema, nausea, meningitis)	RR N/A; mOS 5.8months*; PFS-6 48%	
TP-38 (IVAX) (18)	TGFα + PE38	I	2008	Fatigue, neurological deterioration (seizures, hemiparesis)	RR 13%; mOS 5 months*; PFS-6 N/A	
Tf-CRM107 (19)	Transferrin-DT	II	2003	Cerebral oedema, seizures	RR 35%; mOS 9 months; PFS-6 N/A	Phase 3 studies were aborted or remain unreported (20)
Radioimmunoconjugates						
^125^I-Mab 425 (21)	**IV** murine anti-EGFR (with RT-TMZ)	II	2010	Occasional nausea, flushing, hypotension, skin irritation. Only 4 pts had HAMA	RR N/A; mOS 20.4 months	A sequential cohort with RT alone had mOS 10.2 months
^131^I-81C6 (22)	**LR** murine anti-tenascin (with RT-chemo)	Pilot	2008	Seizures (including status epilepticus), haematological, neurological, infective, thrombotic	RR N/A; mOS 22.6 months	
^131I^-BC2/BC4 (23)	**LR** murine anti-tenascin (with conventional surgery and post-operative treatment)	I/II	1999	Headaches, HAMA reactions	RR N/A; mOS 19 months	Data shown for GBM patients; mOS was 25 months in small volume disease

ADC, Antibody drug conjugate; EGFR, Epidermal growth factor receptor; HAMA, human anti–mouse antibody; LFTs, liver function tests; mOS, median overall survival; mPFS, median progression free survival; N/A; Not Available, RR, Response rate; RT, Radiotherapy; TMZ, temozolomide.

## Newer ADCs in Non-Glioma Therapy

Subsequently, improved ADCs were generated which were characterised by low immunogenicity (usually with chimeric, humanised or fully human antibodies) and more effective payloads (**Table 2**). The success of these newer generation ADCs has been shown in haematological as well as in triple negative breast cancer (TNBC). These include brentuximab vedotin, an anti-CD30, antibody conjugated with monomethyl auristatin E (MMAE), an auristatin payload which disrupts microtubules. This has been shown to improve patient outcomes as consolidation after autologous stem cell transplant in patients with Hodgkin’s lymphoma (37), and the subsequently in combination therapy with chemotherapy in newly diagnosed patients (38). It has also been shown to be effective in patients with CD30-positive T-cell lymphoma (39, 40) and anaplastic large cell lymphoma (41). Trastuzumab emtansine, which carries also carries a microtubule targeting payload, DM1, has shown efficacy in patients with HER2-positive breast cancer and is the first ADC to be approved in solid tumors (42, 43). Other examples of successful ADCs utilising DNA-damaging payloads include inotuzumab ozogamicin with a caliceamicin payload in CD-22 positive ALL (44) and gemtuzumab ozogamicin with a caliceamicin payload in AML (45). Another highly promising class of payloads are is those targeting topoisomerase, such as Sacituzumab govitecan (SG) against Trop 2 and bearing the SN38 payload. SG has shown significant activity in TNBC with improvements in PFS and OS compared to chemotherapy alone (46). In addition, SG has demonstrated activity in intracranial xenograft models and demonstrated activity in patients with recurrent GBM in a single centre pilot study (47).

**Table 2:** Common toxicities associated with antibody drug conjugates.

Payload type	Mechanism of action	Common toxicities
**DM1**	Inhibits tubulin polymerization and causes destabilization of microtubule structures	Thrombocytopenia, fatigue, increased levels of transaminases, anemia, nausea, hemorrhage, abdominal pain, pyrexia, musculoskeletal pain, vomiting, and dyspnea (28, 29)
**DM4**		Elevated transaminases; ocular toxicity (including decreased visual acuity, corneal deposits, keratitis); generalized symptoms (including headache, confusion, fatigue), mucositis (30)
**MMAE**		Infections, nausea, fatigue, diarrhea, peripheral sensory neuropathy, neutropenia, peripheral motor neuropathy, rash, cough, vomiting, myalgia, pyrexia, abdominal pain, arthralgia, pruritus, constipation, dyspnea, loss of weight, and upper respiratory tract infection (31)
**MMAF**		Neutropenia, thrombocytopenia, ocular toxicity (including corneal deposits, keratopathy) (30)
**Calicheamicin**	Binds to the DNA minor groove cleaving the double-stranded DNA	Lymphopenia, skin toxicity, neutropenia, thrombocytopenia; pyrexia, chills, nausea, infection, hemorrhage, fatigue, headache, increased transaminases and hyperbilirubinemia, vomiting, abdominal pain, stomatitis, veno-occlusive disease/sinusoidal obstruction syndrome and diarrhea (32, 33)
**Duocarmycin derivative**		Hypersensitivity, hyperpigmentation (34)
**PBD**		Hypocellular marrow, epistaxis, fatigue, thrombocytopenia, transaminitis, oedema, hypoalbuminemia, dyspnoea (35)
**SN-38**	Prevents DNA unwinding by inhibition of DNA topoisomerase I resulting in irreversible double strand breaks	Thyphilitis, neutropenia, nausea, vomiting (36)

DM1, Mertansine/emtansine; DM4, Ravtansine/soravtansine; MMAE, Monomethyl auristatin E; MMAF, Monomethyl auristatin F; PBD, Pyrrolobenzodiazepine; SN38, Irinotecan metabolite.

## Newer ADCs in Glioma Therapy

In addition to their use in extra-cranial malignancies as described above, these newer ADCs have also been tested in glioma patients with mixed results. As before, targeting EGFR remained highly attractive due to the high frequency of abnormalities in this pathway in high grade gliomas. Furthermore, several highly specific and novel antibodies against EGFR and EGFRvIII had been developed which promised more selective targeting. The monoclonal antibody 806 (mAb806) is a murine anti-EGFR antibody that selectively targets a cryptic epitope of the EGFR which is only exposed under certain conditions, including where wild-type EGFR is highly over-expressed, where there are autocrine loops and/or harbor there are specific mutations which expose the epitope e.g. the EGFRvIII deletion variant. As these conditions are essential tumour restricted, mAb806 and derivative constructs are also tumour restricted with no normal tissue binding. This in this way, there avoid the toxicity typically associated with other systemic EGFR drugs inhibitors (48, 49). ABT-806, the humanized form of mAb806, has shown to be well tolerated and devoid of conventional anti-EGFR toxicities like rash and diarrhoea. Furthermore, biodistribution studies of ^111^In-ABT-806 showed no normal tissue uptake highlighting the tumor-specific nature of mAb806 (49–52). Depatuxizumab mafodotin (Depatux-M) is an EGFR targeting ADC comprising of mAb806 linked to the anti-microtubule toxin monomethyl auristatin F (MMAF). Depatux-M has shown promising *in-vivo* activity in tumor models overexpressing wild type EGFR, *EGFR* amplification, or EGFRvIII mutation (53). Depatuxizumab mafodotin was also found to improve anti-tumour efficacy when combined with radiotherapy and temozolomide in preclinical models (53). The combination was also subsequently confirmed to be safe when tested in a Phase 1 study with newly diagnosed GBM with patients (54), and hence proceed to Phase 3 testing in the INTELLANCE I trial. Unfortunately, the addition of Depatux-M to standard chemo-irradiation with TMZ in newly diagnosed EGFR amplified glioblastoma patients was eventually discontinued for futility (12).

In contrast to the negative results in newly diagnosed patients, anti-EGFR ADCs targeting glioma with EGFR over-expression or EGFRvIII showed clear signals of efficacy in patients with relapsed glioma after chemo-radiation. Depatux-M was evaluated in the randomised phase II INTELLANCE 2 study in patients with EGFR amplified recurrent GBM (55, 56). In this study, the combination of Depatux-M with temozolomide (TMZ) demonstrated a strong trend towards substantial benefit in overall survival compared to the chemotherapy arm (HR 0.71, p=0.062) (57). The benefit of Depatux-M was highest in patients relapsing more than 16 weeks after the start of the last TMZ cycle. No evidence of efficacy in the monotherapy arm was observed in the subgroup with the MGMT promoter unmethylated tumors. These results are given added weight by the results of a Phase I/II study with AMG 595, an ADC comprising a fully human, anti-EGFRvIII monoclonal antibody linked *via* a non-cleavable linker to the maytansinoid DM1. AMG 595 has shown promising preclinical activity in assays including orthotopic murine models (58). In a phase I/II study of AMG 595 in patients with recurrent glioma expressing EGFRvIII (NCT01475006), the most common adverse events were thrombocytopenia (50%) and fatigue (25%); grade ≥ 3 treatment-related AEs occurred in 17 patients (53%). However, it is important to note that two patients had partial responses; 15 (47%) had stable disease, including one patient who was on treatment for 15 months (59). Unfortunately, development of this drug has also been discontinued.

## Future Directions for the Development of ADCs in Glioma

The disappointing results of INTELLANCE 1 has rightly given pause and reconsideration to the role of ADCs in patients with gliomas. It has prompted reconsideration of reason why ADCs may not be suitable for use in patients with gliomas, including the relatively high toxicity when targeting the EGFR family with certain payloads, and the concern that these drugs are unable to penetrate the blood brain barrier to reach glioma tumour cells. One key concern is whether the results of INTELLANCE 1 should be allowed to overshadow the results of INTELLANCE 2 and the AMG-595 study. Much data suggest that recurrent gliomas are different disease from newly diagnosed GBM with changes in its genetic and molecular phenotype (60–66). While the further development of Depatux-M has been terminated by the company, the results of the INTELLANCE 2 study are intriguing about the possible use of this class of ADCs based on the mAb806 antibody particularly when compared to other drugs tested in GBM, such as immunotherapy, which have been universally disappointing in their lack of efficacy (**Table 3**). Formal testing in a phase III would be reasonable but understandably, improved ADCs with a better toxicity profile would be selected if possible. Also, better patient selection is clearly required to identify the subset of patients who clearly have exceptional sensitivity of these ADCs as has been seen with in trials with Depatux-M to date (unpublished data). In a preclinical study, disruption of BBB through the over-expression of vascular endothelial growth factor or avoiding the BBB entirely by direct intra-tumoral injection resulted in improved efficacy of Deptux-M (77). In addition, suppression of EGFR or expression of an EGFR variant lacking the binding epitope and upregulation of compensatory signaling pathways associated with altered EGFR expression and known to function in parallel or downstream from EGFR were identified as potential mechanisms of resistance to Depatux-M.

**Table 3:** Selected trials of therapeutic agents tested in glioma.

Class of drugs	Setting	Trial Description	Target	Phase	NCT	Response rate	OS (months)	Toxicity
**Immunotherapy**								
Nivolumab (67)	Neoadjuvant	Neoadjuvant Nivolumab in Glioblastoma	PD-1	II	NCT02550249	No clinical benefit was substantiated following salvage surgery		NR
	(n = 30)							
(68)								
Pembrolizumab (69)	Neoadjuvant	Neoadjuvant anti-PD-1 immunotherapy in recurrent glioblastoma	PD-1	Pilot	–		13.7	10 patients (67%) in the neoadjuvant group experienced grade 3-4 adverse events likely attributable pembrolizumab
	(n= 35)							
Autologous lymphoid effector cells specific against tumor cells (ALECSAT) (70)	Recurrent	Assess the tolerability and efficacy of ALECSAT in GBM patients (ALECSAT-GBM)		I	NCT01588769	DCR 50%*	NR	5/23 (22%) experienced grade 4/5 toxicity including: pneumonia, respiratory insufficiency, cerebral vascular lesion and general physical health deterioration
	(n = 25)							
CART-cell therapy (71)	Recurrent	Anti- interleukin-13 receptor alpha 2 chimeric antigen receptor (CAR) T-cells	IL13Rα2	I	NCT00730613	NR		
	(NR)							
CART-cell therapy (72)	Recurrent	CMV-specific cytotoxic T lymphocytes expressing CAR targeting HER2 (HERT-GBM)	HER2	I	NCT01109095	DCR 50%	11.1	TRAEs were grade 1-2 and included 3 patients with headache and seizures. No ≥ grade 3 TRAEs reported. No DLT observed
	(n = 17)^±^							
IMA950 multi-peptide vaccine + poly-ICLC (73)	New diagnosis	Trial of IMA950 Multi-peptide Vaccine Plus Poly-ICLC	Human leukocyte antigen (HLA)-A2 restricted peptides	I/II	NCT01920191	DCR 42%	19	Grade 1-2 TRAEs: inflammatory reactions at injection sites (53%), headache (37%), fatigue (63%), and flu-like syndrome (21%)
	(n = 19 16 GBM and 3 grade III astrocytoma)							1 x Grade 4 - interstitial pneumonia due to pneumocystic infection
**Monoclonal antibodies**								
Onartuzumab (74)	Recurrent	Onartuzumab in Combination With Bevacizumab Compared to Bevacizumab Alone or Onartuzumab Monotherapy	c-MET	II	NCT01632228	8.8 (Onartuzumab + Bevacizumab) vs 12.6 (Bevacizumab)		Grade ≥ 3 TRAEs: 38.5% (experimental arm) vs 35.9% (bevacizumab)
	(n = 129)							Experimental arm had higher rates of drug withdrawal + drug interruptions
Tanibirumab (75)	Recurrent	Trial to Evaluate the Safety of TTAC-0001(Tanibirumab)	VEGFR-2	II	NCT03033524	NR	NR	No dose limiting toxicities
	(n = 10)							Cutaneous hemangiomas (83%) - ≤ grade 2 No drug-related G3 or 4 AEs
**Nanoparticles**								
DNX-2401 (tasadenoturev) (76)	Recurrent	DNX-2401 for Recurrent Malignant Gliomas	Oncolytic adenovirus	I	NCT00805376	Group A (n = 25): 20% of patients survived > 3 years		NR
	(n = 37)	Group A (n = 25) - single intratumoral injection of DNX-2401 into biopsy of confirmed recurrent tumor				Group B (n = 12) – NR		
		Group B (n = 12) - intratumoral injection post resection						

*10 of the 25 recruited patients were evaluable.

^±^17 patients included 10 adults and 7 children.

CAR-T cells, Chimeric Antigen Receptor (CAR) T-Cell Therapy; DLT, Dose limiting toxicity; GSC, glioma stem cells; NR, not reported; ORR, Overall response rate; TRAE, treatment related adverse event; VEGFR, vascular endothelial growth factor receptor.

### Improved Drug Delivery Through BBB

One of the main reasons for the ineffectiveness of therapeutic agents intended to target the CNS following peripheral administration is the restrictive nature of the BBB. The BBB is formed by endothelial cells, connected by tight junctions, which continuously interact with surrounding cells like astrocytes, pericytes, and perivascular macrophages, forming the so-called neurovascular unit (78). Primary brain tumors, in particular glioblastoma, cause disruption in the integrity of the BBB as evidenced by the accumulation of gadolinium-based magnetic resonance imaging (MRI) contrast agents within tumor regions. However this disruption is heterogeneous and there is also a clinically significant portion of the tumor with an intact BBB which affects the distribution and efficacy of drugs exposed to this region of the tumor (79). Disrupting the BBB more completely would clearly be useful for treating gliomas with ADCs, amongst other drugs. There has also been recent interest in chemical-induced BBB disruption which has led to increased CNS exposure of ADCs. For example, NEO110, which is a high purity version of the natural monoterpene perillyl alcohol, has been shown to increase the brain delivery of T-DM1 in a mouse model harbouring intracranial HER2+ breast cancer, leading to a significantly greater survival (80). The clinical translation of BBB-disruptors such as NEO110 requires evaluation before such an approach may be considered appropriate for enhancing ADC penetration into the human brain.

In addition to being a physical barrier, the BBB also acts as a biochemical barrier through the function of efflux transporters, such as P-glycoprotein (P-gp) (81) and breast cancer resistance protein (BCRP) (82). While these efflux transporters protect the brain from potentially harmful xenobiotics, they recognise many therapeutics, including a large number of anti-cancer drugs, therefore, limiting their access to the CNS (83). In addition, elimination of the cytotoxic payloads from the cellular cytoplasm by the ATP-binding cassette (ABC) transporters contribute to lower efficacy and resistance to ADCs (26). Increased MDR1 expression has shown to contribute to resistance to auristatin and maytansinoids based ADC analogues, leading to poorer patient outcomes (84, 85). Strategies to overcome drug efflux from cells include using agents that are poor efflux substrates such as hydrophilic compounds or switching from a non-cleavable linker to a protease cleavable and using newer design drugs such as bispecific and biparatopic antibodies can increase cellular internalization (26). Another mechanism that affects the therapeutic effectiveness of ADCs involves defects in the internalization pathway and reduced cell surface trafficking (86). Following internalisation, degradation of ADCs in lysosomes may be impaired by reduced lysosomal proteolytic or acidification function and/or loss of lysosomal transporter expression, resulting in failure of cleavage of cytotoxic payload from ADCs (87). Loss of lysosomal transporter expression, e.g., SLC46A3 has also been reported as a mechanism of innate and acquired resistance to PBD and DM1 bearing ADCs (88). Other potential mechanisms of escape include selection pressure and downregulation of antigens, loss of antigen expression or mutations in antigen as well as presence of ligands for antigens and resistance to ADC and acquired or innate insensitivity to the payload.

With increasing insight into the biology of the BBB and the discovery of novel transporters for trafficking of endogenous compounds, there has been significant interest in attaching natural ligands of these transporter systems to chemotherapeutics to increase their CNS access. One such ligand is angiopep-2, a 19 amino acid peptide targeting the low-density lipoprotein receptor-related protein 1 (LRP1). This has been conjugated to paclitaxel, amongst other anticancer agents. This construct of angiopep-2 and paclitaxel (GRN1005) was shown to be safe and somewhat effective in patients with advanced solid tumours (89), and a subsequent Phase I study showed that GRN1005 had similar toxicity to paclitaxel and some activity in recurrent glioma (90). These techniques were then applied to antibodies, albeit with a modified version of angiopep-2 that also is considered to exploit LRP1 to traverse the BBB i.e. melanotransferrin. Administration of melanotransferrin-trastuzumab conjugate (BT2111) reduced the number of HER2+ breast cancer metastases in the brain (by 68%) with tumours being 46% smaller in BT2111 treated mice relative to control mice (91). To the authors knowledge, there have been no studies where either Angiopep-2 or melanotransferrin have been conjugated to ADCs for the purposes of increasing CNS access, however, based on the results with trastuzumab, it is expected that utilising these shuttle protein approaches should result in increased ADC brain uptake and efficacy.

Lastly, drug penetration into tumours is not just impacted by the BBB. Physico-chemical properties such as tumour volume could be modulated to increase ADC penetration. Larger tumours have increased interstitial pressures, more impaired circulation/lymphatics and increased necrotic areas that limit the ability of ADCs to penetrate the tumours (92–94). Data from the M12-356 Phase 1 study of Depatux-M provides evidence of this problem in glioma patients (95). Preclinical imaging and biodistribution studies showed specific and significantly higher tumor uptake of zirconium-89 labelled Depatux-M (^89^Zr-Depatux-M) in mice with smaller tumor volume *versus* those with larger volumes. Concordantly, mice with smaller tumor volumes at treatment commencement had significantly better growth inhibition and significantly longer overall survival compared to mice with large tumors at treatment commencement. These findings were supported by an analysis of tumor volumes on outcomes in the M12-356 study; patients with large tumors had significantly worse response rates and overall survival. These findings strongly support strategies that would reduce tumor size and/or interstitial pressure to increase efficacy of ADCs in brain tumors (96–98). The tumour microenvironment (TME) in GBM is complex; it is characteristically immunosuppressive and made up of numerous cell types surrounded by a distinctive extra-cellular matrix (99). Dynamic changes in the cellular and metabolic composition within the TME can result in treatment resistant and tumor recurrence (100). Given the role of TME in tumor growth and blood vessel formation, strategies being investigated include targeting antigens of the TME instead of tumor specific antigens as well as overcoming the inherent immunosuppressive effects and making tumors more immune competent (99, 101, 102). Antigens of the TME are likely more accessible and targeting them allows ADCs to accumulate within tumors and release their payload based on TME-specific factors (103, 104). For example, CD25, CD205, B7-H3 are targets found in the TME for which specific ADCs are in clinical development in a number of non-CNS tumour types (105).

### Approaches to Reduce ADC Size/Polarity

The antibody component of an ADC is vital for binding to the desired antigen with high affinity and specificity, maintaining a long half-life and releasing the toxic payload into tumor cells; however its large size presents a physical barrier to efficient extravasation across blood vessel walls and diffusion through tumors (106). This has led to the development of smaller formats as carriers of toxic payloads and include: antibody fragments, peptides, natural ligands, and small molecules (107). Several drug conjugates using smaller targeting domains are being evaluated in clinical trials (NCT02936323, NCT03221400, NCT03486730).

Aptamers are short single-stranded nucleic acids (RNA or ssDNA) that represent a novel imaging and drug-delivery strategy based on their sensitivity and specificity, ease of modification and low immunogenicity (108). Aptamers can be physically conjugated either by intercalating or covalent linking to novel therapeutics such as noncoding RNA or cytotoxic payloads like doxorubicin (109). Recent studies demonstrating the ability of aptamers to cross the BBB and deliver payloads to tumors (110) has resulted in considered interest in the potential role of aptamers in the management of gliomas. To date, several studies have evaluated the role of aptamer-based therapies as well as aptamer-based conjugates (non-coding RNA, nanoparticles, chemotherapeutics) in a number preclinical glioma models (111). The majority of studies targeted EGFR/EGFRvIII while others looked at other GBM-associated proteins including Tenascin-C, EphB2/3 receptors, nucleolin, vascular endothelial growth factor receptor (VEGF), and integrin α5β1 (108, 111). While the results in early preclinical studies are encouraging, further optimization of aptamers with regards to their sensitivity to body-fluid nucleases, CpG toxicity, and stability remain to be optimized.

Nanoparticles are promising carriers for drug delivery to the brain due to their unique characteristics which include their small size and specific and homogenous tumor targeting. Various classes of nanoparticles, including metallic, polymeric and lipid nanoparticles can be readily modified to effectively carry drugs across the BBB. By attaching toxic payloads to nanoparticles, tumour-specific targets expressed in GBM cells and responsible for tumorigenesis can be targeted; examples of these include antigens (i.e., A2B5), differentiation clusters (i.e., CD15, CD33, CD44, or CD133), receptors of cytokines (i.e., interleukin13 receptor), and several proteins (i.e., EGFR, Integrin-a6, α5β3, ανβ3 or L1CAM). An alternative to the classical antibody-drug conjugate is the antibody-mediated delivery of a drug containing nanoparticles. In a recent example, panitumumab/Vectibix was attached to a 400nm nanoparticle (minicell) derived from Salmonella typhimurium in the attempt to deliver an effective dose of doxorubicin to 14 patients with recurrent GBM (112). This study showed that EGFR targeting antibody-coated nanoparticles containing chemotherapeutic drugs could be delivered in recurrent GBM patients.

### ADCs Against Novel Targets

Selecting an appropriate target antigen is a critical step for the success of an ADC. Ideally, the appropriate target antigen should tumor-specific and homogenous in expression. The ideal target is one which is strongly and homogenous expressed on tumor cells, absent on normal tissue and efficient internalisation when bound (113–115). The potency of ADCs is dependent on the ability of the antibody-antigen complex to internalize, release the payload within the target cells and exert the cytotoxic effect. This dependency on antigen expression levels and finite internalization limits the therapeutic potential of ADCs as well as contributes to off-target toxicities (27).Some strategies to overcome the requirement of internalisation include the targeting of non-internalising receptors and extracellular matrix targets. Furthermore, the identification of tumour microenvironment targets also raises the possibility of therapeutic approaches with ADCs which do not target tumor cells alone (8, 26, 115). Novel approaches to developing non-internalization ADCs include diabody-based ADCs against non-internalizing targets and anti-tumor angiogenesis ADC which have shown promise in preclinical studies (116, 117).

The cell surface Notch ligand delta-like 3 (DLL3) inhibits Notch pathway activation and has shown to be expressed on the cell surface of several tumor types including gliomas where DLL3 expression inversely correlated with outcome (118, 119). In brain tumors, DLL3 has been shown to be most intensely and homogeneously overexpressed in *IDH*-mutant gliomas compared to other glioma subtypes (120). Interestingly, in gliomas DLL3 overexpression is not a consequence of *DLL3* mutations or gene amplification (120). DLL3 is not expressed in adult normal tissues (119), making it an attractive therapeutic target. Rovalpituzumab teserine (Rova-T; SC16LD6.5) is an ADC consisting of a monoclonal antibody targeting DLL3, a cathepsin-cleavable linker, and a PBD payload (119). The first-in-human clinical trial of Rova-T in small cell lung cancer (SCLC) demonstrated encouraging activity albeit significant toxicity related to the payload (121). The phase 3 study (TAHOE) in recurrent SCLC however was halted early due futility (122). An active phase 3 trial of Rova-T in the maintenance setting (MERU) is ongoing (NCT03033511). In a phase 1/2 study (NCT02709889) patients with relapsed DLL3-positive (>1% by IHC) advanced solid tumors received Rova-T at 0.2, 0.3, or 0.4 mg/kg every 6-weeks for dose escalation in disease specific cohorts (123). The study enrolled 200 patients including 23 patients with GBM. The recommended phase 2 dose was 0.3 mg/kg q6wk for 2 cycles in all cohorts. The most common adverse events were fatigue, nausea, thrombocytopenia, pleural effusion and peripheral oedema. The objective response rate (ORR) was 11% including one complete response in the GBM cohort.

Increasingly, it is also becoming apparent that targeting the tumour microenvironment may be feasible in glioma and may have advantages over targeting tumours directly. Leucine-rich repeat containing 15 (LRRC15) is a type I membrane protein with low expression in normal tissue but is highly expressed on cancer associated fibroblasts within the tumor stroma as well as directly on cancer cells including GBM (124). ABBV-085 is an ADC composed of an anti-LRRC15 humanized monoclonal antibody conjugated to MMAE *via* a protease cleavable valine–citrulline linker. ABBV-085 has demonstrated significant antitumor activity in multiple LRRC15 cancer-positive models, including GBM. ABBV-085 also showed enhanced activity in combination with other therapies including cytotoxic chemotherapy, radiation, immunotherapy and targeted therapies (124).

Another promising target in the tumour microenvironment is the Eph family. Eph receptor tyrosine kinases and their cell-associated ephrin ligands have been implicated in the growth and progression of a large range of cancers and are increasingly recognized as important therapeutic anti-cancer targets (125). EphA2 and EphA3 are commonly expressed in GBM, including in regions of tumor neovasculature, tumor-associated immune cells, and tumor-infiltrating cells (126), and associated with poorer outcomes in GBM patients (127). MEDI-547 is an ADC comprising an EphA2 targeted monoclonal antibody (1C1) conjugated *via* a non-cleavable linker to the auristatin derivative maleimidocaproyl-monomethyl auristatin phenylalanine (mcMMAF). MEDI-547 displayed encouraging antitumor activity in preclinical models (128) however clinical development was halted due to due to treatment-related adverse events during the early phase studies (129). Despite these results, given the overexpression of EphA2 in many tumor types targeting EphA2 for toxin delivery remains a promising therapeutic strategy. MM-310, an anti-EphA2 immuno-liposome containing docetaxel prodrug has shown superior tumor penetration and anti-tumor activity in a range of xenograft models compared to free docetaxel and significantly with lower toxicity (130). An ADC directed against EphA3, another member of this family, is also being pursued. An ADC based on the IIIA4 mAb, and utilizing the microtubule inhibitor maytansine (IIIA4-USAN), has highly effective in killing preclinical GBM models compared to the naked antibody (131). Similarly, anti-EphA3 bound nanoparticles loaded with the DNA alkylation agent temozolomide showed specific tumor targeting and potent anti-tumor effects in a rat glioma model (132). A phase 1 study of the Ifabotuzumab, the humaneered version of IIIA4, has shown the drug is safe, is able to successfully target the tumour microenvironment in all patients tested but without normal tissue binding (133); an ADC based on Ifabotuzumab is therefore an attractive prospect.

### Novel ADCs With Improved Payloads

Coupled with the above strategies are strategies to utilise newer payloads with increased therapeutic ratios. A number of known issues can adverse impact ADCs. Linkers are an essential interface between antibody and drug payload and are critical in stability, site of conjugation and final drug/antibody ratio (DAR): parameters that impact on toxicity, efficacy and pharmacokinetic properties of ADCs (8, 30). Current linkers may release payloads in the circulation which can lead to off-target toxicity. The development of novel linkers and advances in linker technology is beyond the scope of this review but has been discussed elsewhere in detail (134, 135). In addition, the IgG1 isotype of some of these ADCs can engage the Fc-gamma receptors (FcγR), which can trigger a target-independent, FcγR–dependent internalization in FcγR-positive cells resulting in toxic effects on these untargeted healthy cells (136). In addition, each class of payload often has characteristics toxicities (**Table 2**). A number of newer payloads are being tested. Pyrrolobenzodiazepines (PBDs) are DNA−crosslinking agents that exert their biological activity by binding in the minor groove of DNA with enhanced potent anti-cancer activity compared to auristatins or maytansinoids (137, 138). PBD dimers exhibit significant cell permeability, potentially enabling bystander killing of neighboring tumor cells (139). A number of PBD-conjugated ADCs are being developed and in clinical trials for both solid and hematological tumors (140). For example, ABBV-321 (serclutamab talirine) is a highly selective next-generation EGFR-targeting ADC which incorporates a PBD dimer toxin conjugated to the EGFR-targeting ABT-806 affinity-matured AM1 antibody (10). It is expected that the highly selective nature of ABBV-321 would differentiate it from previously developed antibody PBD conjugates that lack a therapeutic window. In a number of xenograft cell line and patient-derived xenograft tumour models including in GBM, ABBV-321 exhibited potent anti-tumor activity (10). ABBV-321 is currently under clinical investigation in patients with advanced solid tumors (141).

Deruxtecan (DXd) is a potent topoisomerase I inhibitor with a short half-life and ability to elicit a bystander killing effect on neighboring tumour cells indicating low concern in terms of systemic toxicity and importantly may assist in overcoming intratumoral heterogeneity of cancer cells (142, 143). In addition to its by stander effect, DXd is cell membrane permeable and therefore may enter nearby cells, even those without strong HER2 expression, making it effective in low HER2-expressing cancer cells and overcome tumour heterogeneity. Bioconjugation of DXd to a humanized monoclonal antibody specifically targeting HER2 *via* a cleavable tetrapeptide-based linker, made it possible to obtain the conjugate Trastuzumab Deruxtecan (DS-8201a, T-DXd) with a homogeneous DAR of 7.7. Trastuzumab deruxtecan has shown impressive response rates in early phase studies in tumours with high and low HER2 expression as well as HER2 mutant cancers (144, 145). The most common side effects were gastrointestinal and haematological, however potentially fatal adverse event of interstitial lung disease (ILD) was reported in 13.6% of patients in the phase II trial, including four patients who died due to lung injury (146). The mechanism of lung injury is not well understood and predictive biomarkers for response as well as identifying patients at risk of developing toxicity lung injury are required. Another ADC carrying the DXd payload is patritumab deruxtecan which uses the same linker-payload system as trastuzumab deruxtecan and is conjugated to the anti-HER3 monoclonal antibody patritumab. In a phase I study, patritumab deruxtecan demonstrated impressive responses in patients who were heavily pretreated with a median number of four regimens, including EGFR targeting tyrosine kinase inhibitors (147). Notably, its efficacy is observed regardless of the resistance mechanisms for EGFR-TKIs, including C797S secondary EGFR mutation, MET amplification, HER2 mutation, BRAF fusion, and PIK3CA mutation (147).

Lastly, there is on-going interest in utilising new radioisotopes in antibody payload delivery. Both the EphA2 mAb IF7 coupled to Lutetium-177 and then anti-EphA3 antibody IIIA4 linked to an α-particle-emitting Bismuth-213 payload showed therapeutic effect in EphA2 and EphA3 expressing leukemia models (148, 149). In GBM models, treatment with IIIA4 labelled with the β-particle-emitting Lutetium-177 showed dose-dependent tumor cell killing and tumor growth inhibition *in vivo*, compared to unlabelled antibody (150).

### Combinatorial Treatment Approaches to Address Heterogeneity and Resistance

Cancer cells are constantly under strong selection and evolutionary pressures, resulting in the emergence of subclones and heterogeneity in gene expression and antigen expression (151). This in turn can affect ADC efficacy which is correlated with the level of target antigen expression (114). In a phase II study, patients with early-stage HER2-positive breast cancer received six cycles of trastuzumab emtansine (T-DM1), in combination with pertuzumab in the neoadjuvant setting (152). In this study complete pathological response (pCR) was higher in those with HER2 scores of 3+ *versus* 2+ and no pCR was seen among the patients classified as having HER2 heterogeneity, indicating that heterogeneity of target antigen expression is an important factor in patient selection. GBM is characterised by significant heterogeneity even at the single cell level (153). Studies have reported substantial inter- and intra-tumoral variation in the levels of EGFR expression and mutation. Furthermore, EGFR mutations are frequently lost or gained between the initial tumor and recurrence while molecular alterations, such as EGFR amplification, remain persistent unchanged (60). Recently studies have also shown that EGFRvIII can be eliminated from extrachromosomal DNA of tumor cells as a resistance mechanism when tumor cells are treated with EGFR TKIs. However, upon drug removal, EGFRvIII reappears (154). Data from clinical studies suggest that substantial inter - and intra-tumoral variation in the levels of EGFR and EGFRvIII expression and mutation contribute to therapeutic resistance (155, 156).

Combinatorial approaches is one approach to address tumour heterogeneity and resistance in gliomas, and as already demonstrated improved responses and survival rates in other tumor types (157). Dual targeting of both wildtype EGFR and EGFRvIII has been shown to have more effective anti-tumor activity in intracranial murine glioma models than single targeting of either variant alone (158). The anti-EGFR TKI AG1478 has shown to increase mAb 806-reactive dimers on the surface of cells overexpressing EGFR, and the combination has of mAb806 and AG1478 resulted in enhanced anti-tumour activity in xenograft models (159). Furthermore, Orellana et al. demonstrated that stabilizing the inactive kinase conformation with lapatinib correlated convincingly with increased binding of ABT-806 (160), further supporting the approach of targeting both EGFR and its variants.

Combinatorial strategies with ADCs may also effectively address the issue of tumour heterogeneity. Combining ABBV-321 and Depatux-M resulted in greater tumor growth inhibition in an EGFR-overexpressing GBM PDX model compared to either monotherapy treatment (161). It is also possible to incorporate two payloads into an ADC using multi-loading linkers, with improvements in conjugation efficiency and ADC homogeneity. Levengood et al. developed a dual‐auristatin ADCs containing both monomethyl auristatin E (MMAE) and monomethyl auristatin F (MMAF) and showed superior therapeutic benefit in preclinical anaplastic large cell lymphoma models refractory to ADCs comprised of the individual auristatin components (162). Anami et al. developed an ADC composed of anti-HER2 antibody conjugated to the MMAF payload *via* branched linkers and compared with the ADC composed of linear linkers. Their results demonstrated compared to linear linkers, branched linkers were highly stable in the human plasma, having high cell specificity and antigen-binding efficiency, and more significant *in vitro* cell killing potency (163).

Immunogenic cell death of tumor cells induced by cytotoxic compounds used as payloads in ADCs can be potent stimulators of effector T-cell recruitment to tumors and can directly result in dendritic cell activation and maturation (164). Indeed, infiltration of T cells has been observed in tumor biopsy specimens from patients after treatment with ado-trastuzumab emtansine (T-DM1) (165). Based on the induction of antibody dependent cellular cytotoxicity (ADCC) by anti-HER2 therapies, preclinical studies addressed the potential synergistic effect of ado-trastuzumab emtansine (T-DM1) and ICIs. Preclinical studies have shown the combination of HER2-targeting ADCs with ICI resulted in curative responses despite primary resistance to immunotherapy model (166–168). Exploration of tumor specimens from patients enrolled on phase 1 study (M12-356, NCT01800695) (169) has revealed a significant association between T-cell activity and response to ABT-414 treatment (170). This raises the possibility that combination of ADCs with currently approved immunotherapy are very likely to be effective.

### Improved Patient Selection

In order to maximize the therapeutic potential of ADCs and limit exposure of ADCs to patients unlikely to benefit, more sophisticated biomarkers to select patients are needed. The current strategy of identifying patients is based on tumor target expression, which can be challenging due to multiple factors including: tumor heterogeneity, assay sensitivity, and accuracy, potential changes in target expression after multiple therapies, and difficulties in determining threshold levels for target expression that correlate with efficacy (171). In most trials, patients are preselected based target expression on archival tumor tissue. The most commonly employed methodology to determine target expression is by immunohistochemistry (IHC) which does possess limitations including lacks standardisation, reproducibility and, most importantly, correlation with clinical outcome. For example, while antigen expression levels have shown to correlate with response, ADCs have also shown clinical activity in patients with low levels of target antigen expression cancers due to imperfect assays (144, 145). Furthermore, emerging data suggest that selection based on payload sensitivity may add additional value above simply target expression. In the phase 1 study (M12-356, NCT01800695) of Depatux-M in patients with newly diagnosed GBM and recurrent GBM with EGFR amplification, responses in patients with recurrent GBM correlated with EGFR amplification however not all patients with *EGFR* amplification responded (169). Detailed examination of tumors [RNASeq, WES, immunohistochemistry (IHC)] from patients enrolled in the M12-356 trial revealed that mutations for tubulin genes were differentially expressed in responders vs non-responders, with TTLL2, TTLL4, TUBB2A, TUBB2B, TUBG1 and TUBGCP2 mutations (1-3 per tumor) overexpressed in non-responders (172). Preliminary synthetic lethality siRNA experiments have shown that mutations of these genes render sensitive GBM lines resistant to ABT-414 treatment. Furthermore, preliminary analysis of pre-treatment tumor samples with RNASeq has also revealed that responding patients had a higher number of tumor infiltrating lymphocytes (TIL) compared to non-responders, and CD3E expression. This was confirmed by immunohistochemistry analysis of CD3+ cells, with higher CD3+ cells in responding tumors. Preliminary T cell subset IHC analysis has also shown that CD8 cells are more frequently observed in responders, and that CD4 T cells are more abundant in non-responders. These data are highly suggestive of the immune microenvironment of GBM tumors playing a role in the responsiveness of GBM to ABT-414 treatment.

Lastly, molecular imaging by single photon computed tomography (SPECT) or positron emission tomography (PET), or immuno-PET, has been successfully employed to identify target expression, drug distribution and *in-vivo* target delivery and interlesional target heterogeneity (26). The humanized EphA2 mAb 1C1, libelled with ^64^Cu, was used for positron emission tomography (PET) imaging of eight tumor models with different EphA2 expression levels, showing good correlation between tumor uptake and EphA2 expression (125). Imaging of the conformational form of EGFR expressed only on tumors has also been demonstrated in GBM patients with ^111^In-ch806 and ABT-806 (173, 174). The ability to image the target of ADCs has the potential to improve selection of patients and increase therapeutic outcomes in patients with gliomas.

## Conclusion

ADCs have shown to be effective and safe therapies, expanding the armamentarium in several haematological and solid tumors and in many cases transforming treatment paradigms. To date, their potential in glioma patients has not been established. Early ADCs were clearly ineffective due to limitations of early ADCs in general which affected their efficacy in all tumours. More recent ADCs like ABT-414 and AMG-595 clearly show the potential of these drugs in glioma, especially in recurrent gliomas. Clearly, more sophisticated strategies in their use will be needed if we are to make ADCs therapeutically useful for most glioma patients and to improve their therapeutic ratio. A number of such strategies are already available. Improvements in the ADC technology, building on the results of results to date, is clearly needed. These should focus on improvements in selecting targets and payloads. The use of adjunctive strategies is also appealing, seeking to improve drug access to tumours across the BBB and to better select patients. Lastly, combinatorial strategies are likely if we are to substantially improve outcomes in these patients and ADCs could have a major contribution to make in such strategies.

## Author Contributions

All authors contributed to conception and design of the review. All authors contributed to manuscript revision, read, and approved the submitted version.

## Funding

We would like to acknowledge our funding sources including: the Cancer Council Victoria (grant No 1164229), the Victorian Cancer Agency (CRE in Brain Cancer and ECF Fellowship for SP), the Operational Infrastructure Support from the Victorian Government. The support of AMS (National Health and Medical Research Council Investigator Fellowship No 1177837) is also acknowledged.

## Conflict of Interest

The authors declare that the research was conducted in the absence of any commercial or financial relationships that could be construed as a potential conflict of interest.

## Publisher’s Note

All claims expressed in this article are solely those of the authors and do not necessarily represent those of their affiliated organizations, or those of the publisher, the editors and the reviewers. Any product that may be evaluated in this article, or claim that may be made by its manufacturer, is not guaranteed or endorsed by the publisher.
